# Solid Foam Ru/C Catalysts for Sugar Hydrogenation to Sugar Alcohols—Preparation, Characterization, Activity, and Selectivity

**DOI:** 10.1021/acs.iecr.1c04501

**Published:** 2022-02-14

**Authors:** German Araujo-Barahona, Kari Eränen, Jay Pee Oña, Dmitry Murzin, Juan García-Serna, Tapio Salmi

**Affiliations:** † Laboratory of Industrial Chemistry and Reaction Engineering, Johan Gadolin Process Chemistry Centre (PCC), Åbo Akademi University, FI-20500 Turku/Åbo, Finland; ‡ Grupo de Tecnologías a Presión, Instituto de Bioeconomía de la Universidad de Valladolid (BioEcoUVa), Departamento de Ingeniería Química y Tecnologías del Medio Ambiente, Escuela de Ingenierías Industriales, Universidad de Valladolid, 47011 Valladolid, Spain

## Abstract

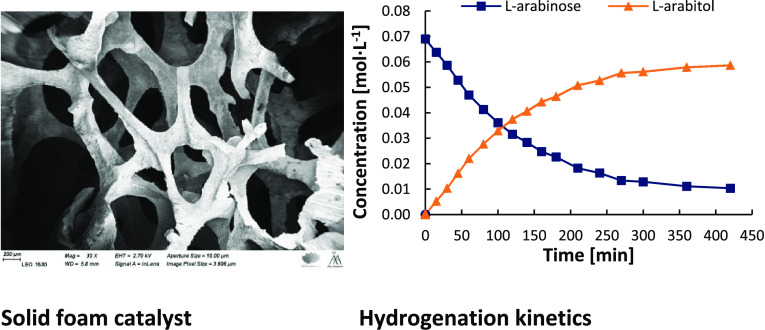

Sugar alcohols are obtained by hydrogenation of sugars in the presence of ruthenium catalysts. The research effort was focused on the development of solid foam catalysts based on ruthenium nanoparticles supported on active carbon. This catalyst was used in kinetic experiments on the hydrogenation of l-arabinose and d-galactose at three temperatures (90, 100, and 120 °C) and two hydrogen pressures (20 and 40 bar). Kinetic experiments were carried out with binary sugar mixtures at different d-galactose-to-l-arabinose molar ratios to study the interactions of these sugars in the presence of the prepared solid foam catalyst. The solid foam catalyst preparation comprised the following steps: cutting of the open-cell foam aluminum pieces, anodic oxidation pretreatment, carbon coating, acid pretreatment, ruthenium incorporation, and *ex situ* reduction. The carbon coating method comprised the polymerization of furfuryl alcohol, followed by a pyrolysis process and activation with oxygen. Incorporation of ruthenium on the carbon-coated foam was done by incipient wetness impregnation (IWI), using ruthenium(III) nitrosyl nitrate as the precursor. By applying IWI, it was possible to prepare an active catalyst with a ruthenium load of 1.12 wt %, which gave a high conversion of the sugars to the corresponding sugar alcohols. The catalysts were characterized by SEM, HR-TEM, TPR, and ICP-OES to interpret the catalyst behavior in terms of activity, durability, and critical parameters for the catalyst preparation. Extensive kinetic experiments were carried out in an isothermal laboratory-scale semibatch reactor to which gaseous hydrogen was constantly added. High selectivities toward the sugar alcohols, arabitol and galactitol, exceeding 98% were obtained for both sugars, and the sugar conversions were within the range of 53–97%, depending on temperature. The temperature effect on the reaction rate was very strong, while the effect of hydrogen pressure was minor. Regarding the sugar mixtures, in general, l-arabinose presented a higher reaction rate, and an acceleration of the hydrogenation process was observed for both sugars as the ratio of d-galactose to l-arabinose increased, evidently because of competitive interactions on the catalyst surface.

## 1 Introduction

The second-generation biorefinery is oriented to the utilization of lignocellulosic biomass created from agriculture, forestry, and alimentary industry, generating chemical compounds from residues.^1,2^ This approach has outstanding advantages such as the wide availability of lignocellulosic materials, which represent 75% of the renewable biomass and the absence of competition for cultivable soil. Lignocellulosic biomass is composed of 40–50 wt % of cellulose (glucose-based polymer linked by β-1,4-glycosidic bonds), 16–33 wt % of hemicelluloses (heteropolymers containing sugar monomers, such as arabinose, galactose, glucose, mannose, and xylose), and 15–30 wt % of lignin (complex cross-linked polymer with coniferyl, coumaryl, and sinapyl alcohols as monomeric units). Elaboration of fuels and chemicals from these materials requires applying thermal, chemical, catalytic, or biological methods to obtain its constituents.^1−3^

Hemicelluloses can be efficiently separated from lignocellulosic biomass with hot water extraction at elevated temperatures, typically 140–180 °C.^4,5^ In the next process step, hemicelluloses are hydrolyzed to sugar monomers and oligomers. Both homogeneous and heterogeneous acid catalysts work in this process, i.e., hydrogen chloride, sulfuric acid, oxalic acid, and formic acid as well as solid cation exchangers, where sulfonic acid is a catalytic agent immobilized to a polymer matrix.^6^

**Figure 1:**
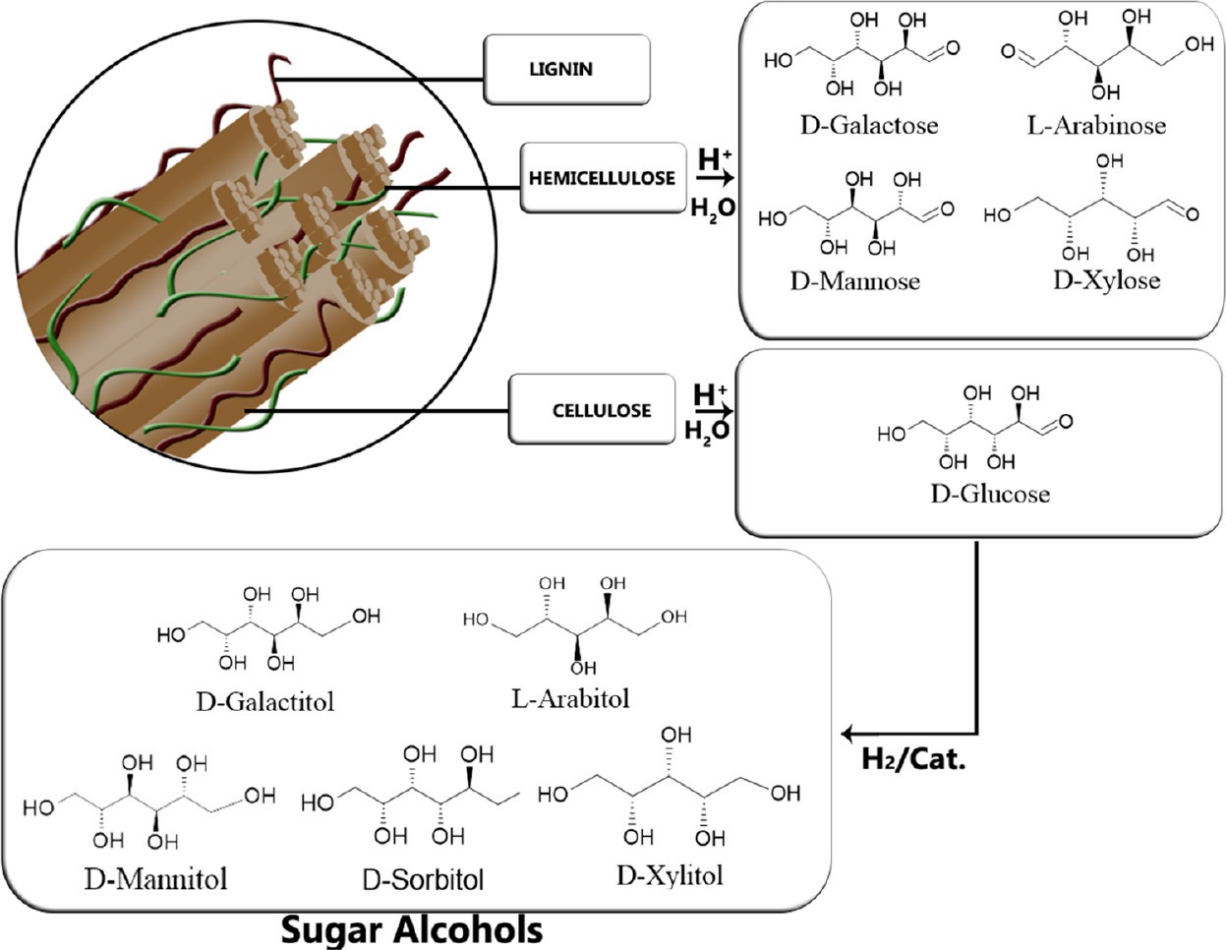
Sugar alcohols from lignocellulosic biomass: catalytic hydrogenation.

From extraction processes combined with chemical treatments, for example, acid hydrolysis, simpler carbohydrates are obtained, such as mono- and disaccharides. Several conversion routes have been proposed to use these compounds as platforms for chemical production. A prime example is glucose from cellulose and starch, which after a reduction process can be transformed into its respective sugar alcohol, sorbitol, and afterward, into polyesters, polyamides, and polyurethanes. Hemicelluloses are present in different biomass sources, e.g., softwood and hardwood, pulping liquors from the paper industry, plant gums, agricultural waste, such as sugar cane bagasse, sugar beet pulp, rice straw, carrot pulp, among others.^6−9^ Sugar monomers like xylose, mannose, rhamnose, arabinose, and galactose can be derived from the major units of hemicellulose present in nature, such as mannans, xylans, arabinans, and galactans. The sugar monomers present in hemicelluloses can be converted to corresponding sugar alcohols as illustrated in Figure 1.

Sugar alcohols are polyols with the general formula (CHOH)*_n_*H_2_ with *n* = 4–6, which are formed by the reduction of the carbonyl group present in the sugar molecules employing either chemical reagents (e.g., sodium borohydride) or molecular hydrogen in contact with a homogeneous or heterogeneous catalyst.^1^ The route based on the use of heterogeneous catalysts is preferred from environmental and technological points of view since it avoids the formation of stoichiometric co-products and facilitates the separation process.^3^

Sugar alcohols find their applications in the alimentary, pharmaceutical, and cosmetics industries. The global market size of sugar alcohols was 3 billion euro in 2019 and is projected to reach 6 billion euro by 2027, exhibiting an increasing rate of 7.75% within 2020–2027.^10^ The main applications of sugar alcohols rely on the alimentary industry, where they are used as healthier alternatives for sucrose due to their sweet taste and low caloric content, especially in the case of xylitol. Sugar alcohols are also widely used in the production of hand sanitizers, which have had a remarkable demand increase since 2020.^10^ It is noteworthy that some studies have shown that sugar alcohols exhibit significant health-promoting effects, such as anticaries and antioxidant activity.^11^

l-Arabinose and d-galactose were the model molecules of this work. These rare sugars can be obtained from arabinogalactan, which appears in large quantities in the Northern Hemisphere in larch species such as *Larix sibirica*. Arabinogalactan consists of β-d-galactopyranose as the backbone with d-galactopyranose and l-arabinofuranose side chains. The average molar ratio of galactose to arabinose is about 6:1, the molar mass is in the range of 20 000–100 000 g/mol, and the average degree of polymerization of around 130–200.^8^

Conventional sugar hydrogenation processes use semibatch reactors operating isothermally (80–150 °C) in the presence of a finely dispersed solid catalyst, in most cases based on sponge nickel often called Raney nickel.^9^ Hydrogen is constantly added to the reactor vessel maintaining the pressure at 10–80 bar. The reaction is usually carried out with an aqueous sugar solution; however, other solvents such as ethanol can be used to improve the hydrogen solubility. Overall, under optimum conditions, high conversions (exceeding 95%) and selectivities toward sugar alcohols are obtained.^12^

Sponge nickel catalysts—often called Raney nickel catalysts—are relatively inexpensive, and they have good activity and reasonably high selectivity. However, they are poisonous, pyrophoric, and subject to deactivation. To surmount these problems, the use of ruthenium catalysts has been ambitioned since it does not dissolve under typical hydrogenation conditions, and it exhibits the highest activity of the conventional catalytic metals: the activity order for glucose hydrogenation is Ru > Ni > Rh > Pd.^9^ Ruthenium catalysts have been intensively studied in recent years for sugar hydrogenation using different support materials such as carbon, alumina (Al_2_O_3_), silica (SiO_2_), titanium dioxide (TiO_2_), magnesium oxide (MgO), and hyper-cross-linked polystyrene. Ru/C catalysts have displayed particularly good performance and stability.^13,14^ Moreover, much effort has been made to develop efficient carbon-supported catalysts that would allow a stable continuous production of sugar alcohols with a special emphasis on structured catalysts, given their advantages over the slurry technology.^15−19^

Structured catalysts consist of regular three-dimensional structures made of ceramics (Al_2_O_3_, cordierite, and SiC), metals (Al, Ni, Cu, Co, or alloy, i.e., stainless steel, Inconel, FeCrAl, NiCrAl, FeNiCrAl), or carbon on which a catalytic material is dispersed.^20−23^ Among the possible configurations used for structured catalysts are monoliths, corrugated open crossflow packings, corrugated closed crossflow packings, knitted packings, fibers, and solid foams.^24^

Structured catalyst materials have features that make them very attractive to be used in chemical reactors, such as a high void fraction, lower pressure drop compared to conventional packed beds filled with pellets, and low flow resistance.^23,25^ These properties have made structured catalysts extremely successful in some commercial applications, particularly in the case of honeycomb monolith catalysts, used in the cleaning of automotive exhaust gases and oxidation of volatile organic compounds.^21^ Thin catalyst layers (≪100 μm) suppress the internal mass transfer resistance in the catalyst pores,^26^ which guarantees high catalyst effectiveness factors and operation under conditions of intrinsic kinetics. The application of structured catalysts enables the shift from batch to continuous technology, which is not easy when catalyst slurries are used.

**Figure 2:**
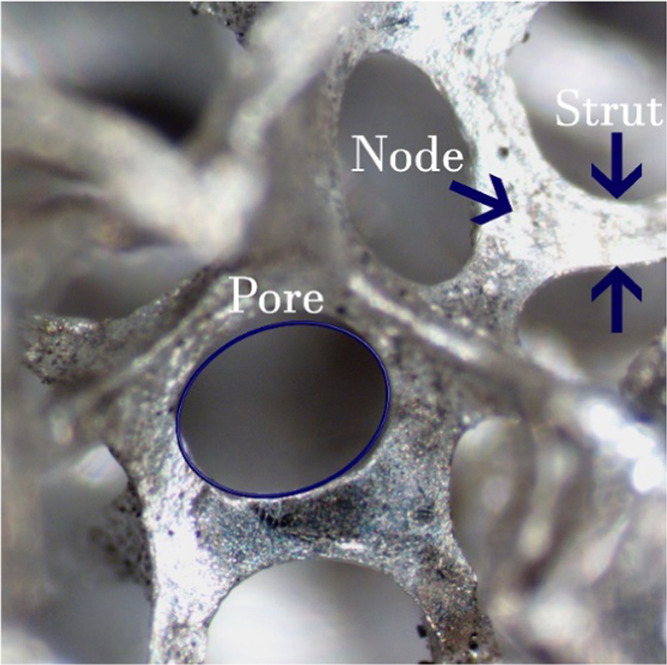
Optical microscope image of an open-cell metallic foam.

Metallic open-cell foam catalysts have been proposed as an alternative to monoliths since they offer higher mass and heat transfer coefficients compared to ceramic monoliths and a lower pressure drop than conventional packed beds.^27^ Open-cell foams are three-dimensional cellular materials made of interconnected solid struts, which enclose cavities (the cells), communicating by windows (the pores), as illustrated in Figure 2. The foam structures provide a disruptive and tortuous flow path and hence an exceptional mixing as well as good heat transfer properties.^27^

However, the absence of micropores in the metallic foams implies a low surface area available for the active phase deposition, but this problem can be solved by coating the foams with appropriate substances that increase the area to take up the catalytic material.^28^ Some authors have investigated the use of furfuryl alcohol (FA) as a carbon coating precursor for structured catalysts.^17−19,29−31^ The use of FA has multiple advantages such as a high carbon yield (around 50%) and reactivity to form resinous carbon compounds.^31^

Carbon coating with poly(furfuryl alcohol) comprises the following steps: controlled polymerization of furfuryl alcohol, immersion of the piece to coat, control of the amount and shape of the polymerized mixture on the surface of the piece, curing (cross-linking), pyrolysis of the formed polymer, activation, and functionalization.^17,29,30,32,33^

It is widely accepted that the predominant product of furfuryl alcohol polymerization under acidic conditions is a linear aliphatic structure of repeating units of poly(furfuryl alcohol) linked by methylene bridges.^33^ On the other hand, the curing degree of the poly(furfuryl alcohol) is highly dependent on the polymerization conditions, i.e., temperature and acid amount; thus, minimal variations of these conditions can lead to a wide range of possible products.^34^

A carbon coating method for metallic open-cell foams was developed by Lali et al.^17^ and developed further by Najarnezhadmashhadi et al.^19^ The authors used furfuryl alcohol as the carbon yielding binder, oxalic acid as the polymerization catalyst, and water as the pore former. The foams were rotated at a constant speed fixed to a stirrer, which prevented the clogging of the pores and allowed a smooth growth of the polymer layers on the struts of the foams.^17^ The method was applied in this work to prepare carbon-coated aluminum foams. Ruthenium incorporation is the next step after preparing the carbon-coated foams. Incipient wetness impregnation (IWI) was used for active metal incorporation on the foams.

The goal of the present research work was to develop a novel open-cell solid foam Ru/C catalyst and to study the catalyst activity and the intrinsic reaction kinetics of the hydrogenation of l-arabinose and d-galactose and their mixtures to sugar alcohols. The following tasks were carried out: development of an effective and reproducible carbon coating method of aluminum foams based on the polymerization of furfuryl alcohol; incorporation of ruthenium on the carbon-coated foams and evaluation of optimal conditions of the incorporation method, characterization of the catalysts, as well performance of kinetic hydrogenation experiments with l-arabinose and d-galactose and their mixtures to explore the product selectivity, reactant conversion, reactant interaction on the catalyst surface, and the influence of pressure and temperature on the reaction rate and product distribution.

## 2 Experimental Section

### 2.1 Overview of Catalyst Preparation

**Figure 3:**
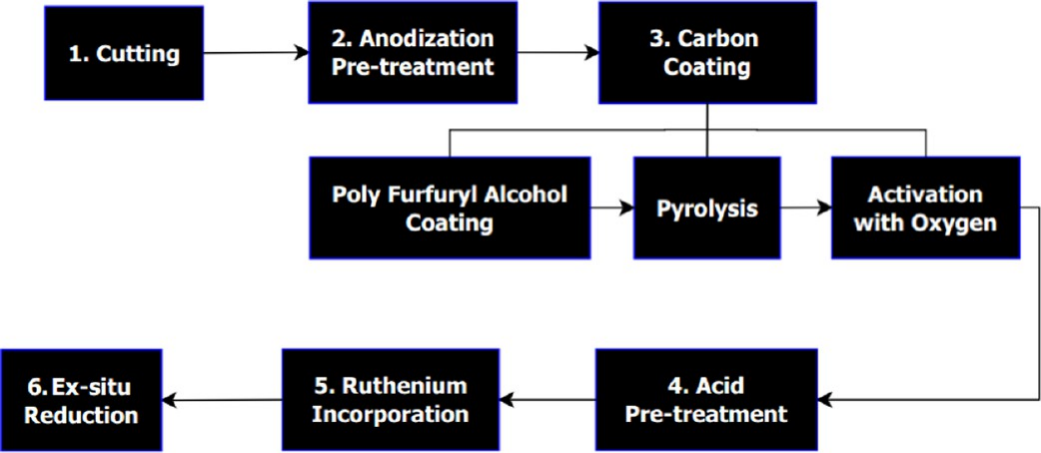
Overview of the solid foam catalyst preparation process.

The catalyst preparation process comprised six general steps: Cutting the open-cell aluminum foam pieces, anodic oxidation pretreatment, carbon coating, acid pretreatment, ruthenium incorporation through incipient wetness impregnation (IWI), and *ex situ* reduction of the catalyst. An overview of the catalyst preparation process is provided in Figure 3.

Ten catalyst batches were elaborated, in which different preparation parameters were tested and several characterization techniques were applied to obtain an efficient catalyst for the kinetic study of the hydrogenation of sugars. Table 1 provides the general information of the prepared catalysts.

**Table 1:** Catalyst Codes and General Information about Preparation

				time [min]					
sample code	initial mass of Al foam^b^ [g]	AOP^a^	rotation speed [rpm]	20–110 °C	110–120 °C	poly(furfuryl alcohol) loaded^b^ [%]	carbon after pyrolysis [%]	carbon after oxygen and acid activation [%]	resulting poly(furfuryl alcohol) coating description
C1	0.481 ± 0.013	no	700	58	29	37.3 ± 0.4	15.2 ± 0.3	11.5 ± 0.4	golden
C2	0.832 ± 0.035	no	700	60	41	33.5 ± 3.3	13.6 ± 1.4	11.5 ± 1.7	golden
C3	0.505 ± 0.011	no	200	55	45	65.4 ± 0.3	45.4 ± 0.2	39.3 ± 0.2	golden
C4	0.463 ± 0.001	no	700	60	34	38.6 ± 0.6	16.7 ± 0.7	14.3 ± 0.7	golden
C5	0.590 ± 0.004	no	200	55	44	75.3 ± 3.9	58.7 ± 1.2	37.6 ± 1.2	foamy and dark
C6	0.589 ± 0.009	no	700	55	45	27.1 ± 0.7	11.7 ± 0.6	7.4 ± 1.0	golden
C7	0.522 ± 0.043	no	700	55	40	46.7 ± 0.8	24.8 ± 1.2	23.0 ± 1.2	foamy and dark
C8	0.551 ± 0.003	no	200	60	50	78.0 ± 1.1	57.2 ± 2.1	53.4 ± 2.1	foamy and dark
C9	0.372 ± 0.004	yes	200	60	50	66.3 ± 0.5	45.9 ± 0.2	45.1 ± 0.2	foamy and dark
C10	0.484 ± 0.032	yes	200	60	50	70.8 ± 1.2	54.7 ± 1.1	53.0 ± 1.2	foamy and dark

a AOP: Anodic oxidation pretreatment.

b The measurement accuracies (±) are based on repeated experiments.

#### 2.1.1 Cutting of Foams

Cylindrical pieces with the dimensions of 33 mm length and 11 mm diameter were cut from a pure aluminum foam sheet with a pore density of 40 PPI (Goodfellow Cambridge Ltd.) using a diamond hole saw bit. The cut foams were sonicated for 15 min in deionized water and for 15 min in acetone and then oven-dried for 2 h at 70 °C and overnight at room temperature.

#### 2.1.2 Anodic Oxidation Pretreatment

To enhance the carbon adhesion to the foams, the surfaces of some aluminum supports were pretreated as follows: A cleaned foam with the above-mentioned dimensions was attached to a thin platinum flat strip using PTFE tape, then connected to the anode (working anode) of a power supply (Autolab PGSTAT100N) with a rectangular 4 cm × 9 cm aluminum plate (the immersed area was 18 cm^2^) connected to the cathode (counter electrode). The anode and cathode were immersed in the electrolyte solution keeping a 2.5 cm distance.

The electrolyte solution consisting of 100 mL of 1.6 M sulfuric acid (Sigma-Aldrich; 96%) and 60 g/L aluminum sulfate hexadecahydrate (Fluka; 98%) was also added to control the dissolution of aluminum during the anodization process.^35,36^ The temperature was set to 40 °C using a thermostat (Grant GR150 GP200) by circulating oil in the jacketed vessel containing the solution. A magnetic stirrer at the bottom of the vessel was utilized to homogenize the mixture composition and the temperature.

A constant electrical current of 2 A was circulated through the system for 1 h, and the voltage was monitored with the General-Purpose Electrochemical System (GPES) version 4.1 software. Thereafter, the foam was taken out from the acid and washed by dipping it in deionized water. The same solution was used to anodize three different foam pieces. The obtained foams were oven-dried at 70 °C for 30 min and then calcined at 600 °C for 4 h.

The required electrical current (2 A) was estimated using the geometrical surface area information and the optimal current density reported by Lali et al.^36^ On the other hand, the time and the electrolyte concentration were chosen by carrying out experiments and evaluating qualitatively the physical stability and homogeneity of the obtained oxide layers.

#### 2.1.3 Carbon Coating

The carbon-coated foam batches consisted of two or three pieces, which were attached to a crossed blade stirrer shaft using thin stainless steel wires and introduced in a 300 mL metallic vessel provided with an electric band heater (Ogden Mighty-Tuff MT-03015-0424). Thereafter, 136.2 g of furfuryl alcohol (Sigma-Aldrich; 98 wt %), 0.42 g of oxalic acid dihydrate (Sigma-Aldrich; 99.5 wt %), and 16.7 g of distilled water were poured into the vessel.

The heating rate of the electrical band was adjusted at 2 °C/min from room temperature (about 20 °C) to 120 °C using a temperature process controller (The CAL 9500P). A Heidolph RZR 2021 mechanical stirrer was utilized to rotate the foams during the polymerization process; two different stirring rates were tested, 200 and 700 rpm.

The mixture under the above-described conditions was kept between 20 and 110 °C within 55–60 min. As the temperature reached 110 °C, the water evaporation began, the liquid viscosity and temperature increased sharply due to the reaction enthalpy; therefore, the automatic heating was turned off and the temperature was adjusted manually to reach 120 °C within 45–60 min in such a way that the water was slowly vaporized. Once the polymerization process was finished, the excess of polymer was removed by centrifuging the foams at 1000 rpm for 5 min.

The polymer-coated foams were pyrolyzed in a furnace (Carbolite CTF 12/100/900) heated at 5 °C/min up to 550 °C and held for 5 h in a nitrogen stream with a flow rate of 1 L/min. Subsequently, the carbon coating was activated in an oxygen stream of 2 L/min, heated from room temperature at 5 °C/min up to 380 °C, and held for 2 h. The experimental conditions and the obtained carbon loads are presented in Table 1.

#### 2.1.4 Ruthenium Incorporation by Incipient Wetness Impregnation (IWI)

Five carbon-coated foams (samples C6 to C10) were pretreated in a 3 wt % nitric acid (Sigma-Aldrich; 70 wt %) solution for 2 h. The acid-pretreated foams were washed in deionized water and oven-dried at 70 °C for 2 h and overnight at room temperature.

Two concentrations of Ru(III) nitrosyl nitrate (diluted in nitric acid solution; Sigma-Aldrich) were tested for the incorporation of ruthenium in the foam catalyst: a 1.4 wt % Ru solution for catalysts C6 and C7, and a 0.6 wt % Ru solution for catalysts C8, C9, and C10.

The precursor solution was dripped to distribute it as homogeneously as possible on the surfaces of the carbon-coated foams using an adequate number of impregnation steps (avoiding overflowing) until reaching the nominal load of each batch as reported in Table 2. For catalysts C6 and C7, the amount of precursor solution per step was approximately 0.10 and 0.25 g for catalysts C8, C9, and C10. After each impregnation step, the foams were dried in an oven at 110 °C for 24 h.

**Table 2:** Ruthenium Incorporation Conditions

batch code	Ru incorporation method	Ru nominal load based on carbon [%]	*ex situ* reduction conditions
C6	IWI	24	450 °C for 2 h
C7	IWI	24	450 °C for 2 h
C8	IWI	4	450 °C for 2 h
C9	IWI	6	450 °C for 2 h
C10	IWI	4	300 °C for 5 h

#### 2.1.5 *Ex Situ* Catalyst Reduction

The *ex situ* reduction of the catalysts was carried out in a furnace (Carbolite CTF 12/100/900), using 1 L/min hydrogen stream under the conditions of time and temperature described in detail in Table 2. The reduction temperature of 450 °C was based on previous experience from Ru/C catalysts.^19^ On the other hand, the temperature of 300 °C was based of TPR measurements gauged in this work with catalyst C10.

### 2.2 Catalyst Characterization Techniques

#### 2.2.1 Scanning Electron Microscopy (SEM)

Scanning electron microscopy (Zeiss Leo Gemini 1530) was used to study the morphology of the anodized aluminum foams, the carbon layer morphology, and the distribution of the carbon-coated catalyst foams prepared under different conditions.

#### 2.2.2 Energy-Dispersive X-ray Analysis (EDX)

Elemental analysis of the Al foams before and after the anodic oxidation process was evaluated by energy-dispersive X-ray analysis (LEO Gemini 1530 with a Thermo Scientific Ultradry Silicon Drift Detector).

#### 2.2.3 High-Resolution Transmission Electron Microscopy (HR-TEM)

High-resolution transmission electron microscopy (HR-TEM) (JEM 1400 Plus Transmission Electron Microscope) was used to measure the Ru particle size distribution of fresh and deactivated catalysts. Electron microphotographs of three random points per sample were obtained, and ImageJ software was utilized to measure 300 particles per micrograph.

#### 2.2.4 Temperature-Programmed Reduction (TPR)

Temperature-programmed reduction (Micromeritics AutoChem 2910) measurements were carried out to study the most active catalyst prepared in this work (C8 and C10). TPR experiments were conducted from 30 up to 700 °C following a temperature ramp of 10 °C/min in a stream of hydrogen and argon (20 mol % hydrogen in argon).

#### 2.2.5 Inductively Coupled Plasma Atomic Emission Spectroscopy (ICP-OES)

The ruthenium content of the catalysts used for the kinetic experiments was gauged by ICP-OES (PerkinElmer, Optima 5300 DV). The carbon coating (0.1 g) of the catalyst samples was digested using a mixture of acids (3 mL of sulfuric acid (Sigma-Aldrich; 96 wt %) + 3 mL of nitric acid (Sigma-Aldrich; 65 wt %)) in a microwave oven prior to the analysis. The leaching of Ru from the catalyst was monitored by analyzing a liquid sample before and after each experiment and determining the Ru concentration via ICP-OES.

#### 2.2.6 Nitrogen Physisorption

The surface areas of catalysts tested with the reaction mixture were investigated through nitrogen physisorption at 77 K (Micromeritics 3Flex-3500). The samples were outgassed for 24 h at 300 °C prior to the analysis; the DFT and BET models were applied to calculate the surface area of the samples and the Barret–Joyner–Halenda (BJH) method was used to estimate pore volume distribution.

### 2.3 Experiments in Semibatch Reactor

**Figure 4:**
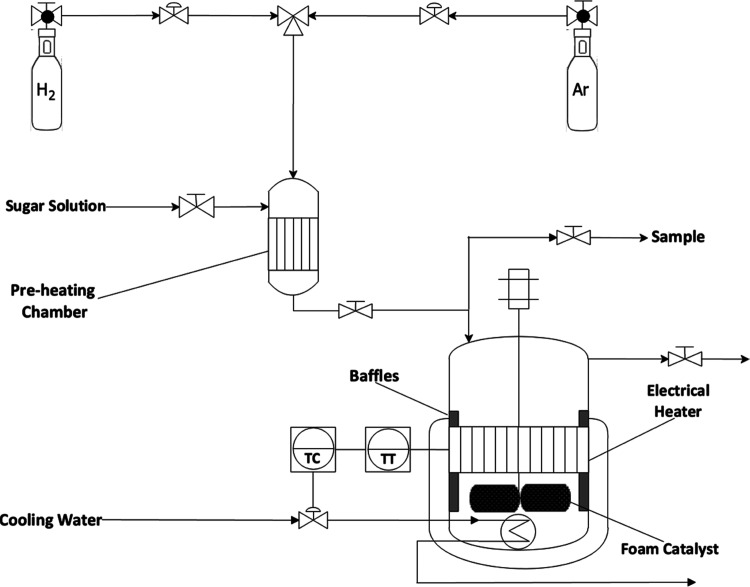
Overview of the setup for sugar hydrogenation experiments.

The kinetic experiments were carried out in a 0.3 L laboratory-scale semibatch reactor (Parr 4561) provided with baffles, a sampling line with a sintered filter (7 μm), a heating jacket, a temperature and stirring rate controller (Parr 4843), a cooling coil, a pressure display module (Parr 4843), and a bubbling chamber. Two foam catalyst pieces were mounted at the endpoint of the mechanical agitating shaft to work as the stirrer during the experiments. The equipment flowsheet is shown in Figure 4.

A set of systematic kinetic experiments were carried out with l-arabinose and d-galactose at three temperatures (90, 100, and 120 °C) and two hydrogen pressures (20 and 40 bar) in the presence of catalyst C8, using a 0.13 M sugar solution.

To study the interaction of the sugars during the hydrogenation reaction, a series of experiments was conducted using binary mixtures of d-galactose and l-arabinose in the presence of catalyst C10. The reaction conditions were 120 °C and 20 bar, varying the initial molar ratio of d-galactose to l-arabinose (G:A ratios, 0.5, 1, and 5).

Prior to the kinetic experiments, the reactor was purged with argon and hydrogen, the foam catalyst was reduced *in situ* for 2 h at a 5 bar pressure of hydrogen and 120 °C. After reducing the catalyst, 130 mL of sugar solution was pumped to the bubbling chamber and purged with argon and hydrogen for 15 min each; then, the temperature was set to the desired value, the hydrogen pressure was adjusted, and the hydrogen-saturated solution was injected into the reactor. Hence, the experiments started exactly under the desired conditions of temperature and hydrogen pressure. A stirring rate of 600 rpm was used in all of the experiments. Samples were withdrawn from the reactor to measure the concentrations of the reagents and products.

The concentration analysis of the sugars and sugar alcohols was conducted using a high-performance liquid chromatograph (Hitachi Chromaster HPLC) equipped with a refractive index (RI) detector (Hitachi 5450 RI Detector). A Biorad HPX-87C carbohydrate column was used with 1.2 mM CaSO_4_ solution (0.5 mL/min flow rate) as the mobile phase, the temperature of the oven was 70 °C, and an injection volume of 10 μL was utilized. The calibration data are shown in the Supporting Information. The sugar conversion and product selectivity toward sugar alcohols were calculated using eqs 1 and 2, respectively.
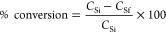
1
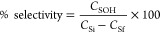
2

## 3 Catalyst Preparation Results and Discussion

**Figure 5:**
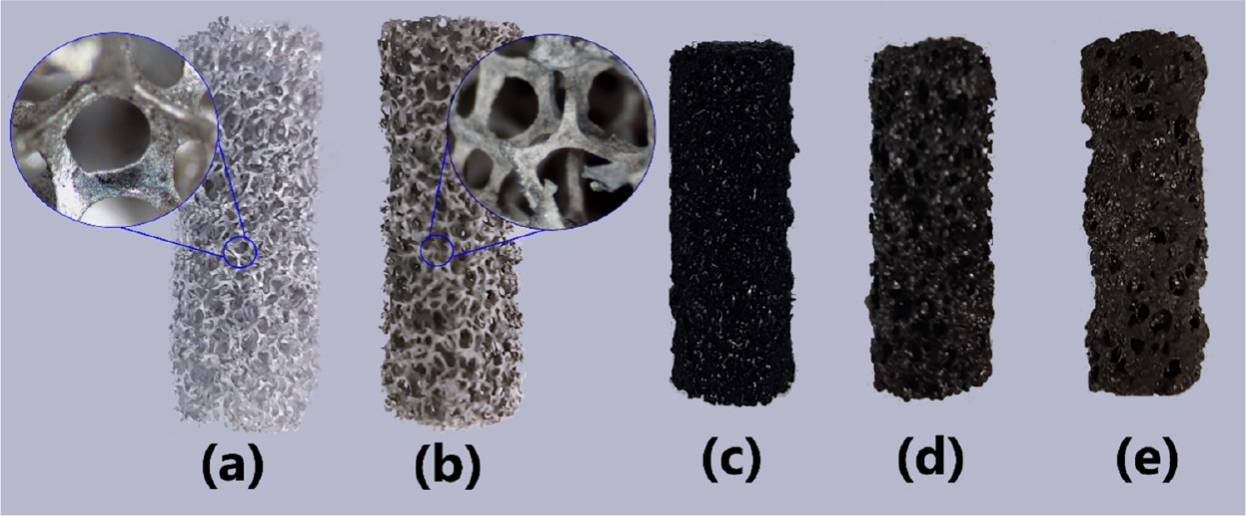
Changes in the open-cell foam catalyst through the preparation stages: (a) Al-untreated foam, (b) anodized Al foam, (c) foam coated with poly(furfuryl alcohol), (d) pyrolyzed/oxygen-treated carbon-coated foam, and (e) carbon-coated, Ru-impregnated, and reduced catalyst.

Figure 5 shows the open-cell foam catalyst at the successive preparation stages. The shrinkage of the piece after the heat treatment stages is noticeable.

### 3.1 Anodic Oxidation Results

An anodic oxidation pretreatment was performed to generate surface roughness on some aluminum foam samples to improve the carbon cohesion in the coating step. The acid resistance of the used platinum strip allowed total immersion of the foam piece and good contact throughout the process.

The voltage recorded in all samples increased to a maximum in less than 0.5 s and decreased to a constant value of 3 V. This indicates that an oxide layer is formed at the beginning of the process followed by the growth of pores on the surface, and finally an equilibrium is established between the formation and dissolution of the oxide.^37,38^

**Figure 6:**
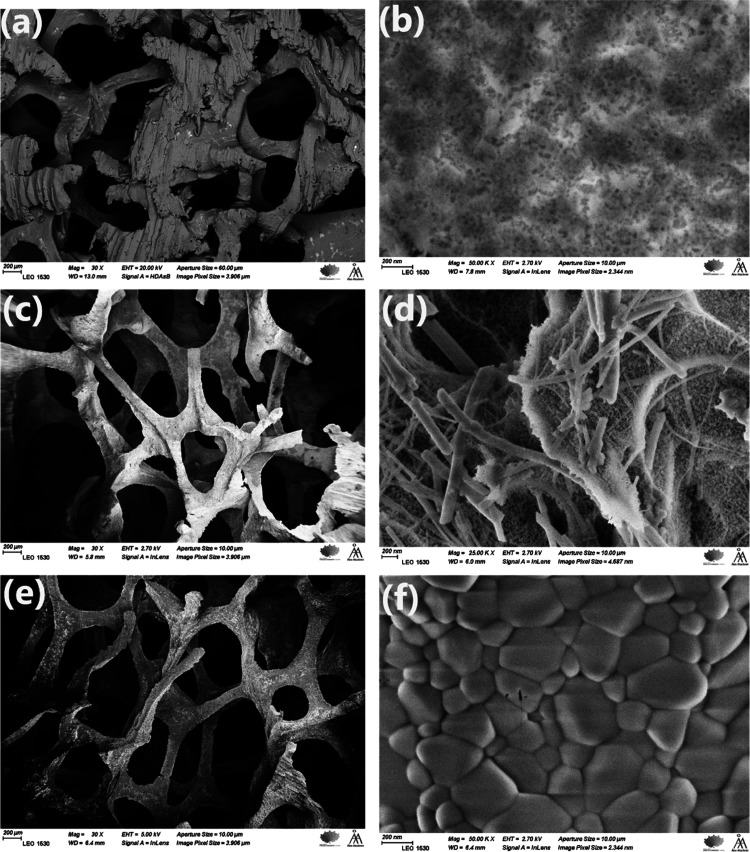
SEM micrographs of the oxide texture generated in catalyst C10. (a) Untreated foam (30X), (b) untreated foam (50 kX), (c) anodized foam (30X), (d) anodized foam (50 kX), (e) anodized and calcined foam (30 kX), and (f) anodized and calcined foam (50 kX).

After the anodic oxidation, the glossy silver color of the untreated aluminum foam pieces changed to a gray matte color, implying a well-distributed oxide layer as can be seen in Figure 5. The SEM images (Figure 6) of the surface textures at different stages of the anodic oxidation process revealed that the surface was changed from a mainly smooth texture to be covered by fiber-shaped features in the case of the anodized sample, and by semiregular hexagonal nanopores (with an average size of 220 nm) in the case of the anodized and calcined sample. These pores of a hexagonal arrangement are typical for anodic aluminum oxide.^37,38^

The differences between the micrographs before and after the calcination demonstrate the need for such a treatment to obtain a more uniform pore pattern and to eliminate surface sub-holes. This effect is ascribed to the diffusion of the ambient oxygen and the aluminum from the substrate through the existing aluminum oxide layer, which combine to form additional alumina, suggested also by the remarkable increase of the oxygen content after the calcination step as reported in Table 3. On the other hand, the increase in the content of other minority elements (S, Mg, Si, Fe) can be ascribed to the presence of impurities in the used sulfuric acid.

**Table 3:** Elemental Analysis (EDX) of Aluminum Foam during the Different Anodic Oxidation Stages (Sample: C10)

stage	Al [wt %]	O [wt %]	Fe [wt %]	S [wt %]	Si [wt %]	Mg [wt %]
untreated foam	99.33 ± 0.47				0.67 ± 0.17	
anodized foam	66.59 ± 0.30	28.72 ± 0.19	0.12 ± 0.05	2.20 ± 0.06	2.08 ± 0.10	0.29 ± 0.030
anodized/calcined foam	41.63 ± 0.16	46.20 ± 0.38	0.44 ± 0.05	6.04 ± 0.07	1.79 ± 0.08	3.89 ± 0.08

### 3.2 Carbon Coating Results

The carbon coating of the aluminum foams was carried out by a controlled polymerization of furfuryl alcohol, followed by a pyrolysis step, and carbon activation under an oxygen stream. Table 1 shows that the residence time between room temperature and 110 °C influenced the carbon load, which is consistent with previous observations about this kind of coating process.^17,19^

Under the experimental conditions, two kinds of polymeric coatings were obtained. The first one was a foamy and dark-colored material formed when there was a sudden increase in temperature and water vaporization after reaching 110 °C, while in the absence of these effects, the result was a golden-colored and less viscous polymer. In general, the carbon made from the dark foamy polymer exhibited better properties to be used as catalyst support: higher surface area, more homogeneous coverage, and better resistance to acids.

Despite that the exact reaction mechanism and products obtained in the polymerization of furfuryl alcohol remain uncertain, it is widely accepted that under acid conditions, the main product is a linear aliphatic structure of repeating units of poly(furfuryl alcohol) linked by methylene bridges, produced by the condensation of the OH groups. As the branching and cross-linking of the linear poly(furfuryl alcohol) take place, the mixture becomes darker and more viscous, and the water vaporizes due to the exothermic character of these phase reactions, creating cavities on the polymer, which enables it to become a good active carbon precursor.^39^

**Figure 7:**
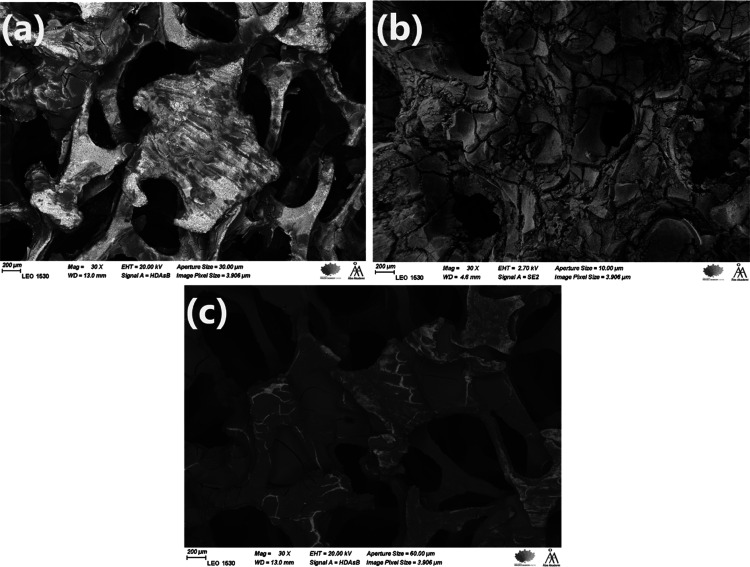
Surface structure of a carbon-coated foam substrate: (a) C2 (∼12 wt % carbon), obtained from golden-colored poly(furfuryl alcohol); (b) C8 (∼50 wt % carbon), obtained from foamy dark poly(furfuryl alcohol); and (c) C10 (∼50 wt % carbon), preanodized and obtained from foamy dark poly(furfuryl alcohol).

As shown in Figure 7, the carbon coating obtained from the less cross-linked poly(furfuryl alcohol) looks inhomogeneous and has a considerable amount of uncovered areas compared to the carbon from the foamy polymer. Additionally, the anodized foams presented a carbon coating with fewer cracks and improved the cohesion due to the surface roughness.

Another significant parameter identified was the rotation speed used in the experiments. The ruthenium incorporation experiments indicated that a carbon content exceeding 40 wt % was required to deposit enough active metal on the support. Thus, a rotation rate of 200 rpm was used, resulting in higher carbon loads under similar polymerization conditions (Table 1).

### 3.3 Nitrogen Physisorption Results

The specific surface areas of three catalysts (C4, C5, and C8) with different carbon content are displayed in Table 4. The nitrogen adsorption isotherms (Figure S1 in the Supporting Information) displayed an open hysteresis loop, as reported in previous studies for poly(furfuryl alcohol)-derived activated carbon; this observation suggests the presence of narrow micropores or bottleneck pores.^39^ On the other hand, the distribution of BJH pores showed that the highest density for pores with a size equal to or less than 1.3 nm (Figure S2 in the Supporting Information).

**Table 4:** Comparison of the Surface Area of Prepared Catalysts at Different Carbon Loads

catalyst	carbon content	total carbon mass (two foams) [g]	BET specific surface area [m^2^/g]	DFT specific surface area [m^2^/g]
C4	14.3 ± 0.7	0.150	52.91	66.67
C5	37.6 ± 1.2	0.73	38.69	75.11
C8	53.4 ± 2.1	1.27	43.06	83.62

### 3.4 Ruthenium Incorporation

Incipient wetness impregnation was used to incorporate Ru on the surface of carbon-coated foams to increase the active metal content of the catalyst. Two concentrations of Ru(III) nitrosyl nitrate were investigated: 1.4 wt % Ru and 0.6 wt % Ru; the amount of precursor solution per step was established as the maximum liquid volume that could be uptaken by the support without overflow.

Two fundamental aspects for the preparation of this kind of catalyst were confirmed as a result of the IWI tests; the carbon obtained from the foamy poly(furfuryl alcohol) exhibited superior adsorption of the precursor solution compared to the carbon from the golden-colored polymer, and the presence of nitric acid in the precursor solution represents a risk for the aluminum structure if the carbon load is insufficient.

**Figure 8:**
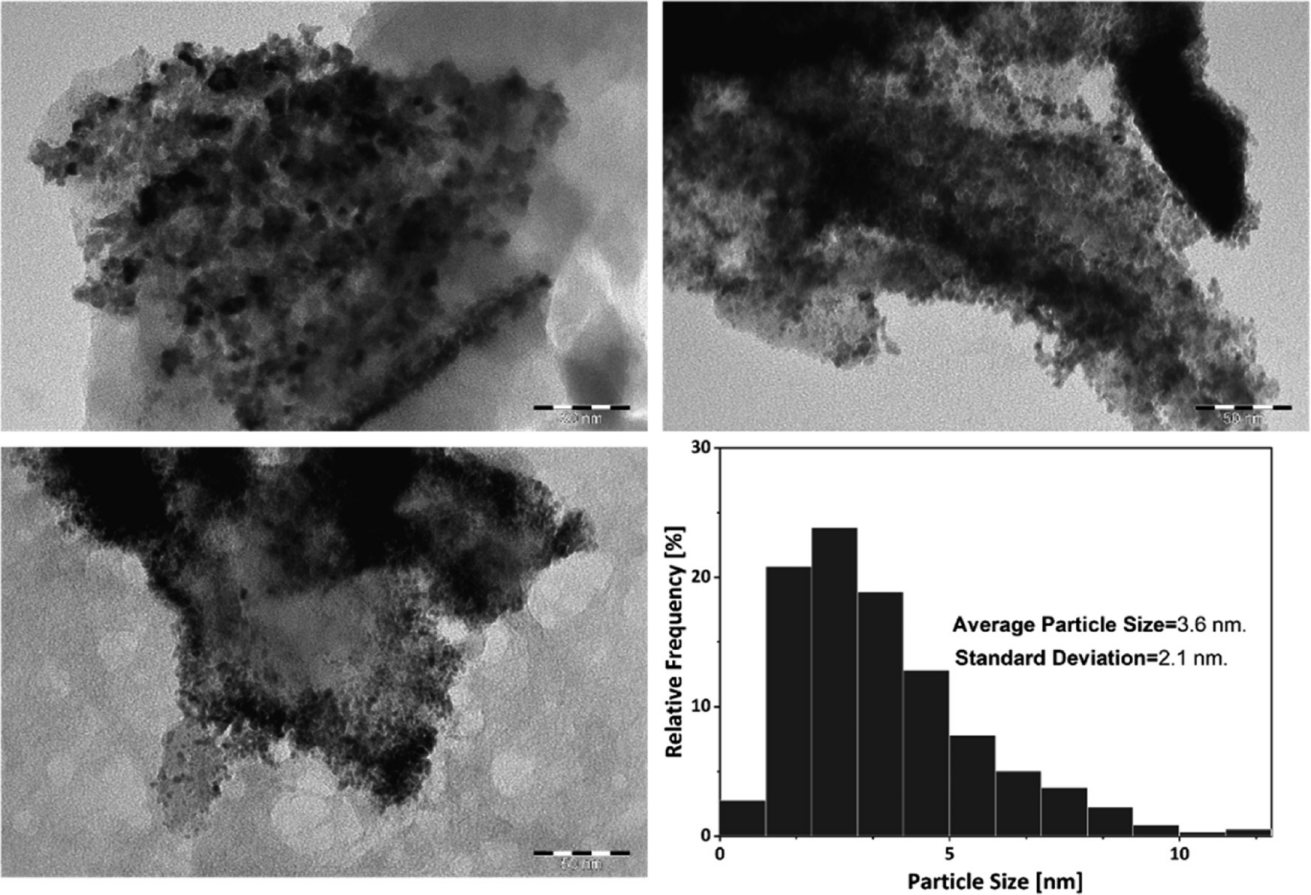
TEM images of catalyst C10 and Ru nanoparticle size distribution.

Therefore, the most active catalysts (C8 and C10-) obtained in this work were elaborated using supports with a high carbon content (∼50%), a precursor solution with a concentration of 0.6 wt % Ru, and a nominal load of 4 wt % Ru based on carbon, yielding a 1.12 wt % of Ru content with an average nanoparticle size of 3.7 nm, and 70% of the particles smaller than 4 nm as shown in Figure 8.

### 3.5 Effect of Reduction Conditions and Catalyst Durability

**Figure 9:**
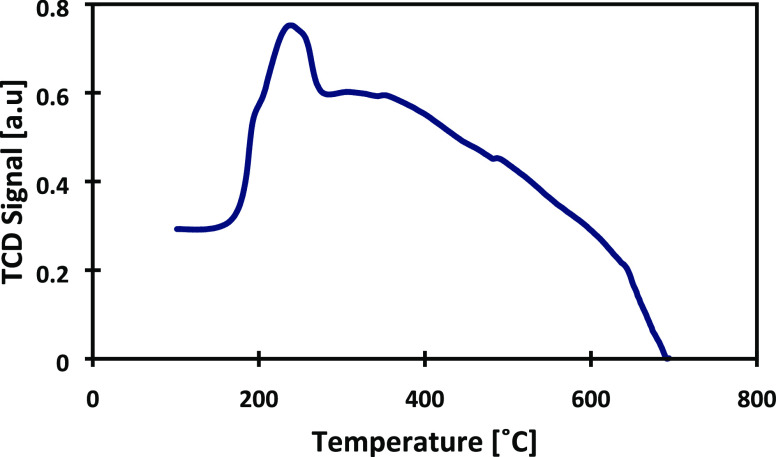
Hydrogen-TPR profiles of catalyst C10 (before *ex situ* reduction).

Under the reduction conditions of 450 °C and 2 h, catalyst C8 displayed an increase in its activity after every subsequent experiment; this behavior can be ascribed to the presence of unreduced Ru oxides, which are reduced during the reaction, forming more active metallic sites Ru^0^.^40−43^ The temperature-programmed reduction (TPR) measurements displayed in Figure 9 were conducted with the catalyst C10 to establish more adequate reduction conditions. A single hydrogen consumption peak appeared at 245 °C, attributable to the reduction of ruthenium oxides.^44,45^ Therefore, the new reduction temperature conditions were set at 300 °C and a prolonged time of 5 h (temperature ramp 3 °C/min) for the catalyst C10, for which no reactivation was observed.

**Figure 10:**
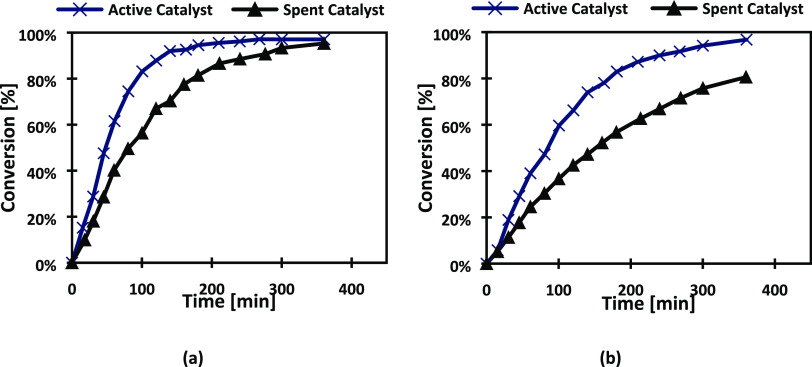
Deactivation of catalyst C8 during hydrogenation of (a) l-arabinose and (b) d-galactose at 120 °C and 20 bar.

On the other hand, after 96 h of use, equivalent to 14 subsequent experiments, the catalyst C8 presented a considerable deactivation for the hydrogenation of both l-arabinose and d-galactose as demonstrated by successive experiments displayed in Figure 10. Therefore, H_2_-TPR, ICP-OES, and TEM measurements were conducted to investigate the possible reasons for deactivation.

**Figure 11:**
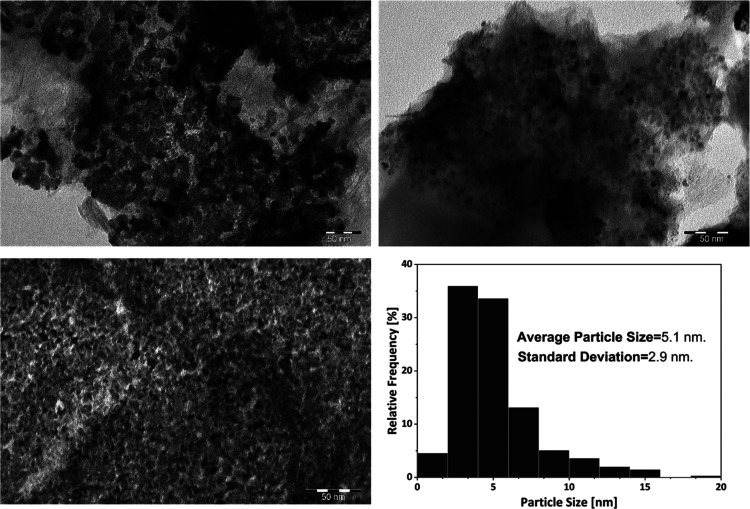
TEM images of catalyst C8 after 100 h of use and particle size distribution.

Figure 11 shows the TEM micrograph of the spent catalyst. A substantial agglomeration of nanoparticles had taken place, and the average size increases from 3.6 nm (Figure 8) to 5.1 nm. Some authors have reported the agglomeration of Ru nanoparticles after hydrogenation reactions of sugars.^46,47^

Previous studies have suggested the formation of Ru(OH)*_x_* species during liquid-phase reactions in the presence of water.^15,33,48^ Nevertheless, the TPR measurements carried out with the spent catalyst did not show any significant hydrogen consumption peak within the temperature range of 400–500 °C associated with the reduction of these species. However, no Ru leaching under the experimental conditions was detected (detection limit: <0.03 mg/L). Simakova et al.^14^ have found that the rates of l-arabinose and d-galactose hydrogenation on Ru/C catalysts are highly influenced by the metal cluster size, with a maximum turnover frequency at 3 nm (approximately the particle size of the fresh catalyst), and that activity decays rapidly as the size increases, indicating that the increased particle size in our catalyst is the reason for deactivation.

In general, the prepared catalyst exhibited good selectivity, activity, and stability similar to^49^ and even higher than other Ru/C catalysts described in the literature.^47^ In that sense, Ru/C foam catalysts are a promising technology to be used in the continuous production of sugar alcohols due to their thin catalyst layer that suppresses the internal mass transfer resistance (≪100 μm),^26^ as well as the disruptive and tortuous flow path provided by the foam structure that gives excellent mixing properties and lower pressure drops compared to the conventional slurry technology.^23,25^

## 4 Kinetic Results and Discussion

### 4.1 Hydrogenation Results of Individual Sugars

#### 4.1.1 Product Selectivity and Reactant Conversion

Individual sugar hydrogenation experiments were conducted at 20 bar and 90, 100, and 120 °C on the prepared Ru/C foam catalyst (catalyst C8). The reaction conditions were selected in such a way that the external and internal mass transfer limitations were suppressed. A high stirring speed was applied to eliminate gas–liquid and liquid–solid mass transfer resistances, and concerning the internal mass transfer resistance in catalyst pores, operation in the regime of intrinsic kinetics was ensured by comparing the reaction and diffusion rates according to the criterion of Weisz and Hicks.^50^ Because the change of the liquid volume during the reaction is minor, the volume of the reaction medium was considered constant.

The overall selectivity toward the sugar alcohols (l-arabitol and d-galactitol) was higher than 98% in all of the cases, while the conversion ranged from 53 to 97% depending on the temperature. l-Arabinose presented higher reactivity than d-galactose, as can be seen in Table 5.

**Table 5:** Selectivity and Conversion in the Hydrogenation of l-Arabinose and d-Galactose after 6 h of Reaction at 20 bar and Different Temperatures (Hydrogenation of Individual Sugars)

	d-galactose		l-arabinose	
temperature	conversion [%]	selectivity [%]	conversion [%]	selectivity [%]
90	53.16	100	84.69	100
100	73.16	99.35	96.53	99.82
120	96.75	98.23	97.05	99.74

The yield of the byproducts was negligible (1–5%) in all of the experiments and dependent on the operation conditions; higher pressures and higher temperatures resulted in the formation of more byproducts, which could be detected by inspecting the chromatograms.

#### 4.1.2 Temperature and Pressure Effects

**Figure 12:**
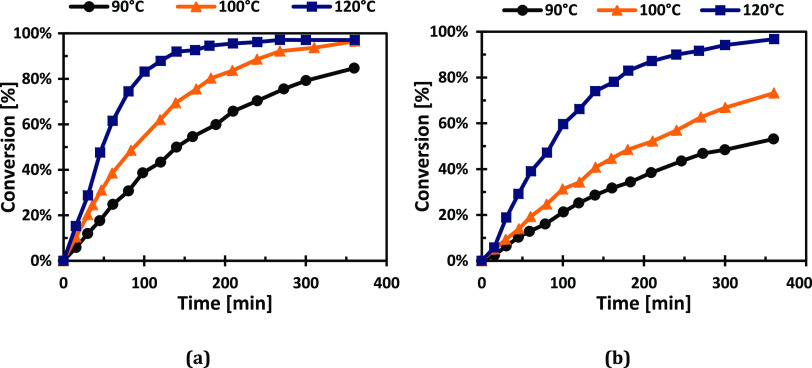
Effect of temperature on the hydrogenation rates at 20 bar for (a) l-arabinose and (b) d-galactose.

The reaction temperature had a very significant influence on the hydrogenation rate for both sugars, as can be seen in Figure 12.

**Figure 13:**
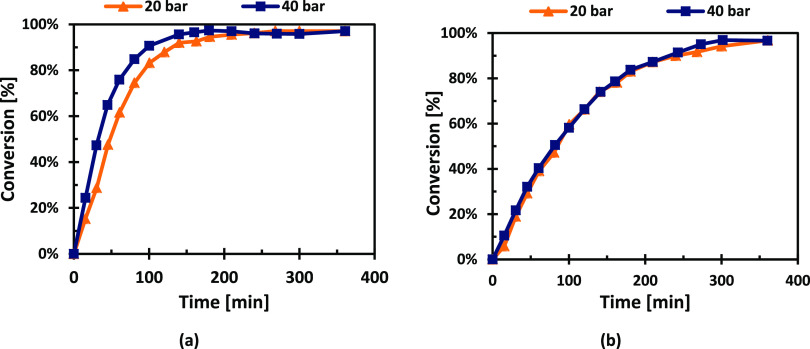
Effect of the hydrogen pressure on the hydrogenation rates at 20 bar for (a) l-arabinose and (b) d-galactose.

On the other hand, the effect of hydrogen pressure on the reaction kinetics was rather minor, as illustrated in Figure 13. Although the effect is weak, the effect of pressure at other temperatures was not studied due to the catalyst deactivation.

**Figure 14:**
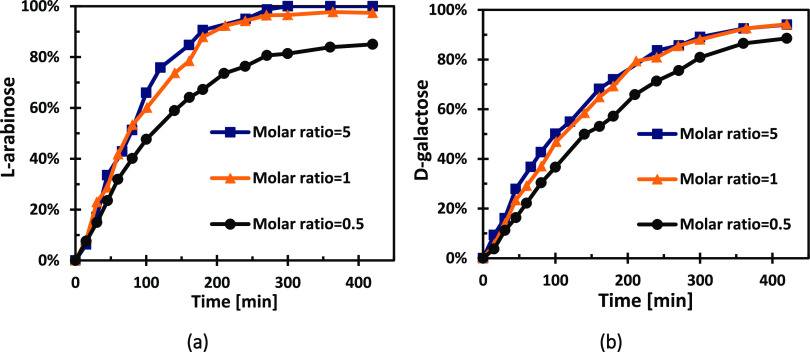
Effect of the d-galactose-to-l-arabinose molar ratio on the hydrogenation rates at 120° of (a) l-arabinose and (b) d-galactose.

These results are very consistent with the observations reported by Sifontes Herrera et al.^51,52^ who carried out several sugar hydrogenation experiments in the presence a Ru/C powder catalyst. It was found that the temperature has a strong effect on the hydrogenation rate, while the hydrogen pressure has a minimal effect, with the extreme case of d-galactose that exhibited almost an invariant behavior with respect to the hydrogen pressure. This insignificant effect of hydrogen pressure indicates strong adsorption of hydrogen on Ru surface. Because of this extremely minor effect of hydrogen pressure on the rate, it is not possible to give a final conclusion on the adsorption state of catalytically active hydrogen. Both hypotheses, dissociative and nondissociative adsorptions, give rather similar rate expressions with respect to the hydrogen pressure. Concerning the role of the sugar adsorption, Figures 12–14 give a clear indication: in the very beginning of the experiment, the concentrations are almost straight lines as a function of time, but they get bent as the reaction progresses, i.e., the reaction order with respect to the sugar is shifted from a low value (close to zero) toward first order at high conversions. This kind of behavior is very characteristic for sugar hydrogenation, as confirmed by previous investigations.^13,14,50−52^ From the reaction rates, the activation energies were determined using logarithmic plots, ln (rate) vs reciprocal absolute temperature (1/*T*). The apparent activation energies were estimated to be 56 kJ/mol for l-arabinose and 68 kJ/mol for d-galactose.

### 4.2 Hydrogenation of Binary Sugar Mixtures

To study the interaction of the sugars during the hydrogenation reaction, a series of experiments were conducted using binary mixtures at 120 °C and 20 bar, varying the molar ratio of d-galactose to l-arabinose (G:A ratios: 0.5, 1, and 5). As in individual sugar experiments, high sugar conversions (85–99%) and high sugar alcohol selectivities (95–99%) were obtained and the yield of byproducts was almost undetectable after 6 h of reaction. Regarding the effect of the molar ratio, d-galactose exhibited an increase in the reaction rate as the ratio of d-galactose in the mixture was higher, which is an expected result that can be ascribed to the presence of other sugar competing for the same active sites on the catalyst. However, l-arabinose displayed an acceleration in the reaction rate with an increase of the d-galactose-to-l-arabinose ratio, as can be seen in Figure 14. Although counterintuitive, this effect has been previously observed in competitive catalytic reactions, where the addition of a component (d-galactose in our case) leads to an increase in the reaction rate. This has also been observed by Sifontes Herrera et al.^52,53^ for d-galactose-l-arabinose mixtures; so, in general, the increase in the concentration of l-arabinose retards the hydrogenation rate of both sugars which compete for hydrogen. The foam structure as such seems not to have a direct impact on the mixture hydrogenation since a very similar effect has been observed for Ru/C catalyst powder.

Noteworthy, these results demonstrate the possibility of carrying out the direct hydrogenation of sugar mixtures, such as those obtained from the selective hydrolysis of arabinogalactan, the hemicellulose, resulting in a mixture with an approximate molar ratio of d-galactose to l-arabinose of 6:1.

## 5 Conclusions

A selective and durable open-cell solid foam catalyst based on ruthenium nanoparticles was developed, characterized, and tested for the hydrogenation of l-arabinose and d-galactose and their binary mixtures to the corresponding sugar alcohols.

A carbon coating method based on the polymerization of furfuryl alcohol (FA) was successfully applied to prepare carbon-coated aluminum foams. The cross-linking of poly(furfuryl alcohol) was identified as a relevant parameter to obtain a homogeneous carbon layer with desired properties for the catalyst support. The temperature control and water evaporation during the polymerization of FA were extremely important to generate a cross-linked foamy polymer as the base for an active carbon support for ruthenium nanoparticles. Surface roughness was induced on some aluminum foams prior to the carbon coating through anodic oxidation, which improved the cohesion and homogeneity of the carbon layer as revealed by SEM images.

The ruthenium incorporation was made by incipient wetness impregnation (IWI) using Ru(III) nitrosyl nitrate as the precursor. The carbon load on the foams and the concentration of the precursor solution were identified as the most important parameters for the ruthenium incorporation. Under the found optimal conditions, it was possible to obtain a catalyst with distributions of small-size Ru particles, with an average nanoparticle size of around 3 nm and 1.12 wt % Ru content. The correct establishment of the *ex situ* reduction conditions is essential to obtain a stable catalyst; this was evidenced for some prepared catalysts that presented an increase in their activity in each consecutive experiment caused by an insufficient reduction time. Thus, TPR measurements were conducted, and the hydrogen exposure time was prolonged, establishing new *ex situ* reduction conditions at 300 °C and 5 h.

The hydrogenation of l-arabinose and d-galactose on the Ru/C foam catalyst yielded a very high selectivity toward sugar alcohols (≥98%) and conversions in the range of 53–97%, depending on the temperature. The influence of the reaction temperature on the reaction rate was strong, while the hydrogen pressure effect was rather minor, especially in the case of d-galactose. Regarding the binary sugar mixtures, l-arabinose exhibited a higher rate than d-galactose in the mixtures and an acceleration in the hydrogenation of both sugars was observed as the ratio of d-galactose to l-arabinose was increased, evidently as a result of competitive interaction of the sugars.

After about 100 h of use, some catalyst deactivation was observed. TEM micrographs of the spent catalyst revealed that substantial agglomeration of the ruthenium nanoparticles took place, resulting in the increase of the average size from 3.6 to 5.1 nm, suggesting that this phenomenon is the main cause of deactivation.

In general, the prepared catalyst exhibited good selectivity, activity, and stability similar^49^ and even superior to other Ru/C catalysts described in the literature.^47^ In that sense, Ru/C foam catalysts represent a promising technology to be applied on the continuous production of sugar alcohols due to their thin catalyst layer that suppresses the internal mass transfer resistance (≪100 μm),^26^ as well as the disruptive and tortuous flow path provided by the foam structure that gives excellent mixing properties and lower pressure drop compared to the conventional slurry technology.^23,25^

## Supporting Information Available

Nitrogen adsorption data; HPLC calibration information; and kinetic results of sugar hydrogenation (PDF)
