# Supplementary material for: Solid Foam Ru/C Catalysts for Sugar Hydrogenation to Sugar Alcohols—Preparation, Characterization, Activity, and Selectivity
Source: Ind Eng Chem Res. 2022 Feb 14;61(7):2734–47. doi: 10.1021/acs.iecr.1c04501 (PMC8883585; doi:10.1021/acs.iecr.1c04501)
Supplement: Supplementary file 1 — ie1c04501_si_001.pdf [file ie1c04501_si_001.pdf]

Supporting Information

Solid foam Ru/C catalysts for sugar hydrogenation to sugar alcohols – Preparation, characterization, activity, and selectivity

German Araujo-Barahona[1, 2], Kari Eränen[1], Jay Pee Oña[1], Dmitry Murzin[1], Juan García-Serna[2], Tapio Salmi[1]

[1]Laboratory of Industrial Chemistry and Reaction Engineering, Johan Gadolin Process Chemistry Centre (PCC), Åbo Akademi, FI-20500 Turku/Åbo Finland

[2]Grupo de Tecnologías a Presión, Instituto de Bioeconomía de la Universidad de Valladolid (BioEcoUVa), Departamento de Ingeniería Química y Tecnologías del Medio Ambiente, Escuela de Ingenierías Industriales, Universidad de Valladolid, 47011, Valladolid, SPAIN.

Corresponding author, tapio.salmi@abo.fi

Nitrogen physisorption isotherms

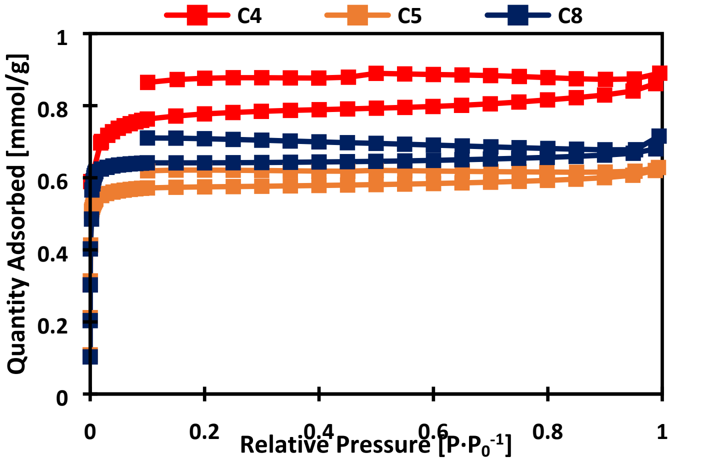

Figure S1. Nitrogen physisorption isotherm at 77K for tested catalysts.

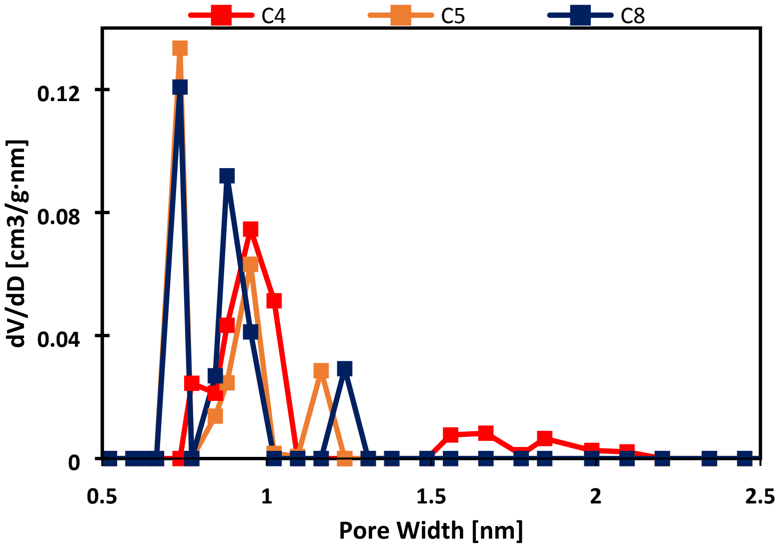

Figure S2. Pore distribution of tested catalysts using the BJH method.

High-performance liquid chromatography

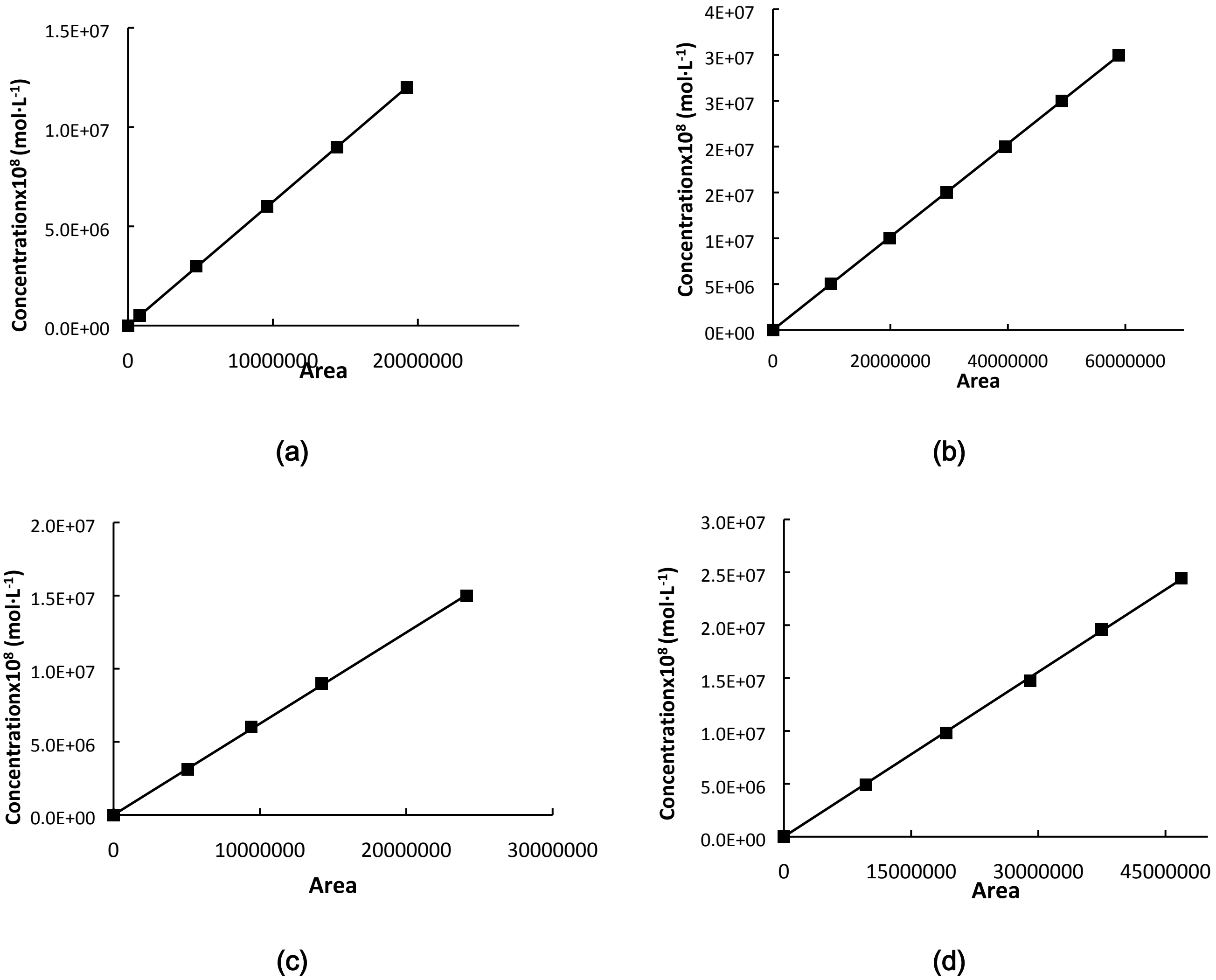

Figure S3. HPLC calibration curves for sugars and sugar alcohols: (a) L-arabinose, (b) D-galactose, (c) L-arabitol, (d) D-galactitol.

$$C_{Sample} = a \cdot A_{HPLC} \cdot 10^{-8} \qquad (S1)$$

Table S1. HPLC calibration factor and retention times for sugars and sugar alcohols.

Component	a	Retention Time [min]
L-arabinose	0.6244	16.557
D-galactose	0.5077	14.387
L-arabitol	0.6245	22.193
D-galactitol	0.5188	26.123

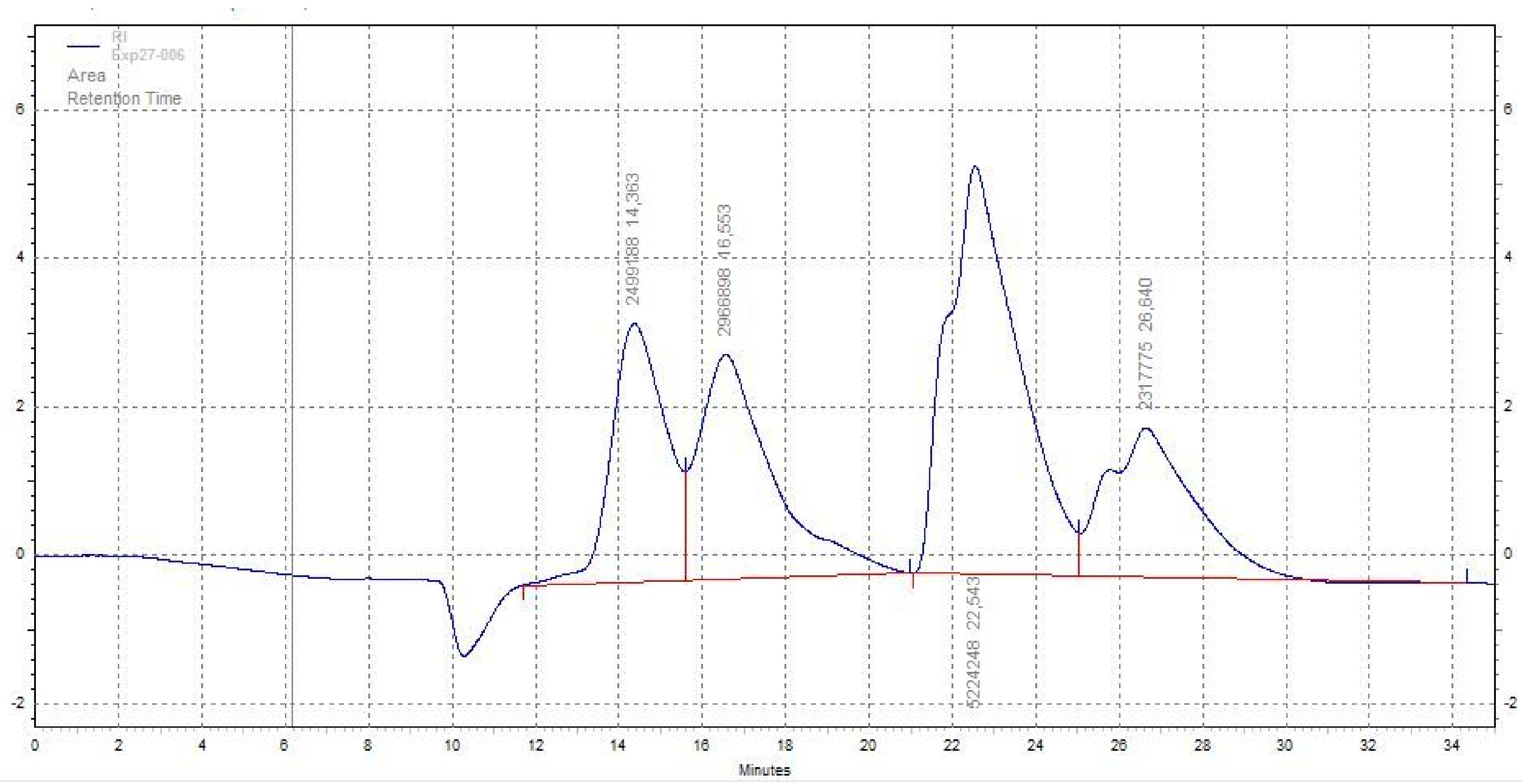

Figure S4. Chromatogram displaying reagents and products during sugar mixtures hydrogenation experiments.

Kinetic results

Individual sugar experiments

Results from hydrogenation experiments of L-arabinose and D-galactose at 20 bar and different temperatures (120˚C, 100˚C and 90˚C).

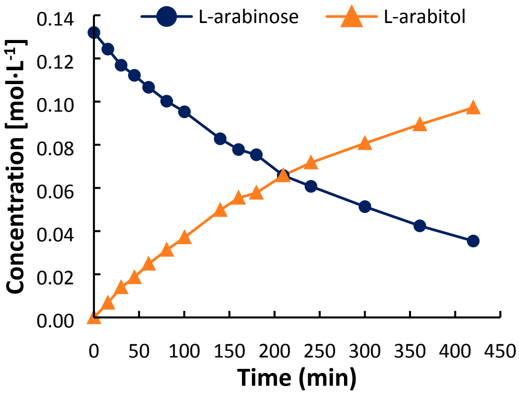

a)

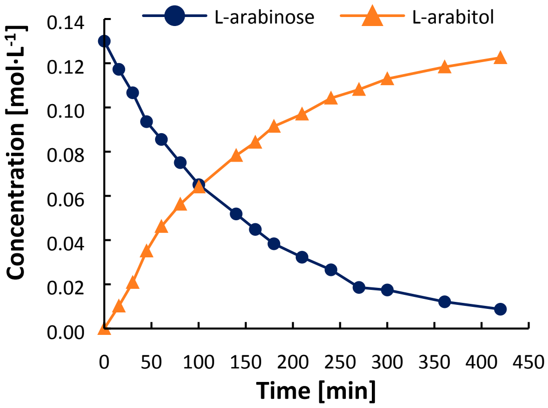

b)

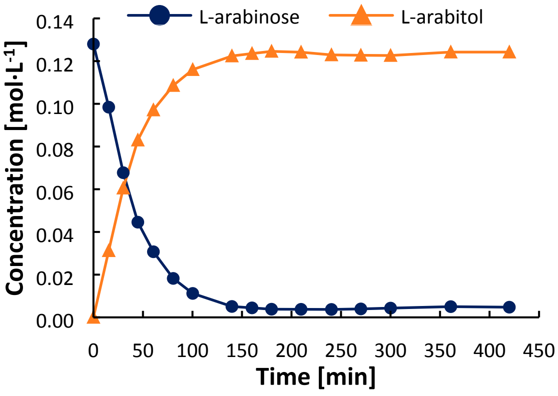

c)

Figure S5. figure Hydrogenation of L-arabinose—0.13M at 20 bar varying temperature a)90˚C, b)100˚C and c) 120˚C

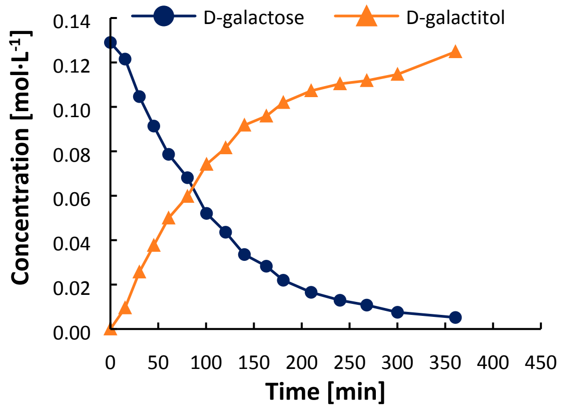

a)

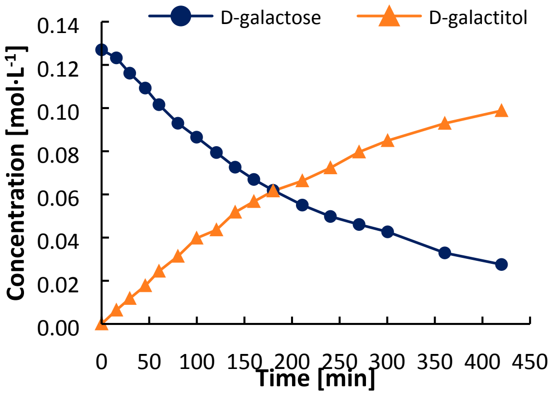

b)

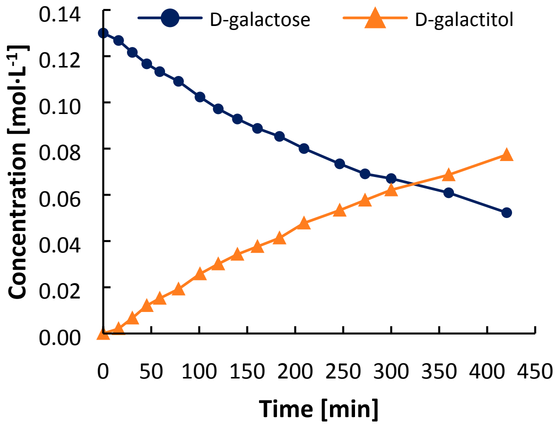

c)

Figure S6. Hydrogenation of D-galactose—0.13M at 20 bar varying temperature a)120˚C, b)100˚C and c) 90˚C

Mixture experiments

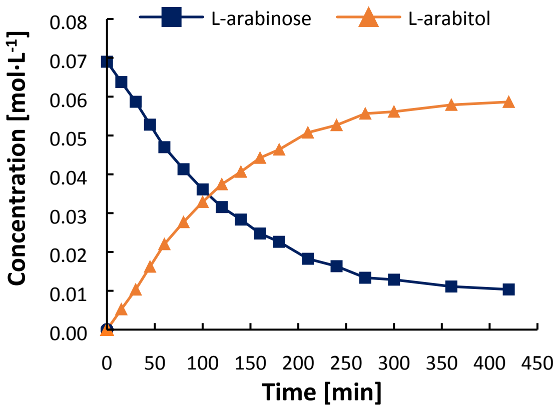

(a) **(b)**

Figure S7. Hydrogenation of sugar mixtures hydrogenation results at 120˚C and 20 bar. Ratio=0.5: (a) L-arabinose, and (b) D-galactose.

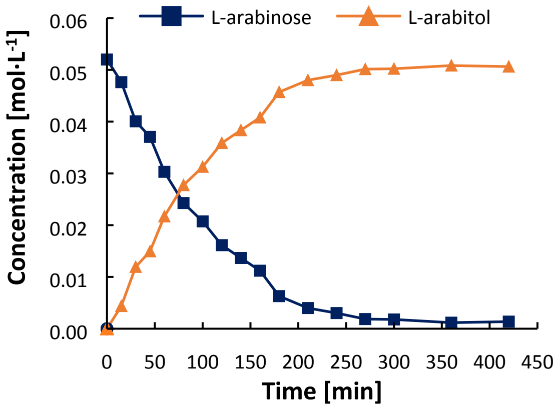

(a) **(b)**

Figure S8. Hydrogenation of sugar mixtures hydrogenation results at 120˚C and 20 bar. Ratio=1: (a) L-arabinose, and (b) D-galactose.

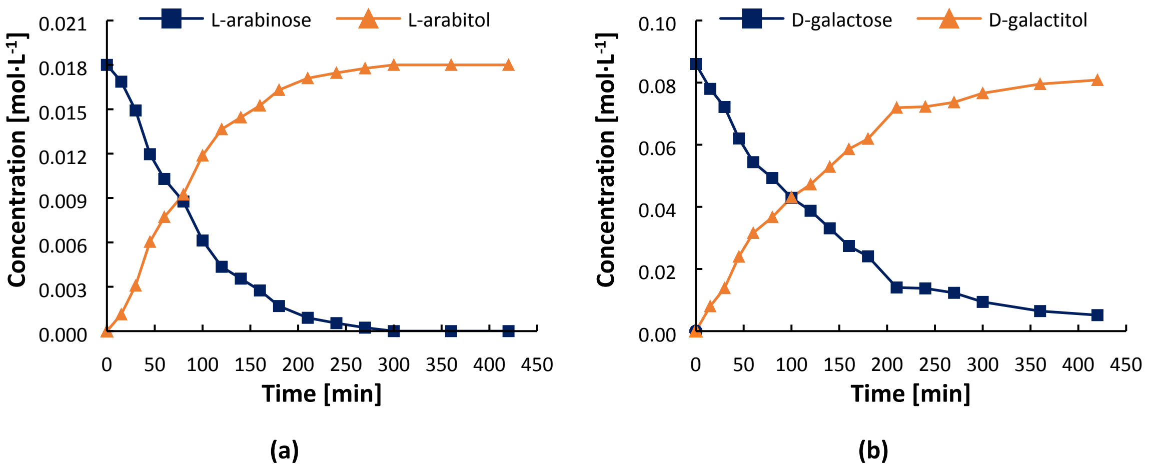

Figure S9. Hydrogenation of sugar mixtures hydrogenation results at 120˚C and 20 bar. Ratio=5: (a) L-arabinose, and (b) D-galactose.
